# β-Caryophyllene gradients act as ecological filters shaping microbial life-history strategies via iron competition

**DOI:** 10.1093/ismejo/wrag181

**Published:** 2026-07-10

**Authors:** Ruolan Li, Yuhui Li, Qifan Yang, Yinuo Ding, Gaofei Jiang, Yan Xu

**Affiliations:** Agro-Environmental Protection Institute, Ministry of Agriculture and Rural Affairs, Tianjin 300191, China; Agro-Environmental Protection Institute, Ministry of Agriculture and Rural Affairs, Tianjin 300191, China; Agro-Environmental Protection Institute, Ministry of Agriculture and Rural Affairs, Tianjin 300191, China; Agro-Environmental Protection Institute, Ministry of Agriculture and Rural Affairs, Tianjin 300191, China; Jiangsu Provincial Key Lab for Organic-based Fertilizer Creation and Soil Health Manipulation, Key Lab of Organic-Based Fertilizers of China, Jiangsu Collaborative Innovation Center for Solid Organic Wastes, Educational Ministry Engineering Center of Resource-Saving Fertilizers, Nanjing Agricultural University, Nanjing 210095, China; Agro-Environmental Protection Institute, Ministry of Agriculture and Rural Affairs, Tianjin 300191, China

**Keywords:** volatile, ecological filter, microbial adaptive responses, life-history traits, iron competition

## Abstract

Plant-emitted volatile organic compounds (VOCs) are increasingly recognized as key mediators of plant–microbe interactions, yet how they shape microbial community assembly and adaptive strategies remains unclear. Here, using the widespread plant sesquiterpene β-caryophyllene as a model VOC, we demonstrate that plant volatiles drive dose-dependent ecological filtering and microbial adaptation, governed by life-history trade-offs and resource availability. Soil microcosm experiments revealed that low concentrations of β-caryophyllene selectively enrich competitive bacterial taxa, whereas high concentrations favor ruderal, fast-growing opportunists, restructuring communities along a competitor–stress tolerator–ruderal axis. Focusing on representative taxa, *Bacillus subtilis* and *Pseudomonas aeruginosa*, we show that these contrasting ecological outcomes are underpinned by divergent physiological, metabolic, and transcriptional reprogramming. Across both taxa, low-dose β-caryophyllene consistently induces siderophore biosynthesis, identifying iron availability as a central integrator of volatile perception and response. Experimental manipulation of iron reprogrammed growth, carbon utilization, and antibiotic resistance, gating whether β-caryophyllene functions as a signal that primes competition or as a stressor that selects for ruderal traits. Together, our work offers critical insights into the VOC-mediated microbiome management for ecological restoration and sustainable agriculture.

## Introduction

Plant-emitted volatile organic compounds (VOCs) can transcend the spatial constraints of the rhizosphere, diffusing through air and soil as signaling molecules to reshape soil communities across larger spatial scales [1–3]. Primarily comprising terpenoids, phenylpropanoids, fatty acid derivatives, and related compounds, VOC emissions are dynamically regulated in response to biotic and abiotic stresses [4–6]. The prevailing paradigm holds that root exudates are central to mediating plant–microbe communication and in shaping the dynamics of soil ecosystems [7–9]. However, the unique functions and ecological significance of VOCs have been largely overlooked, highlighting a critical knowledge gap. Unlike soluble root exudates, which are primarily localized in the rhizosphere, VOCs can diffuse over long distances through air and soil, enabling them to influence both aboveground and belowground ecosystems simultaneously [10–12]. This broad spatial reach allows VOCs to mediate complex plant–plant and plant–microbe interactions across larger scales, enabling their role as versatile signaling molecules across diverse ecological contexts [13]. Numerous studies have demonstrated that plant-emitted VOCs serve dual roles, which involved in initiating defense mechanisms within the emitting plant and establish cross-species early warning networks among neighboring plants [14–16]. A well-documented example is the emission of methyl salicylate, which functions as an airborne signal to alert nearby plants of pathogen invasion or the presence of alien species [15]. These roles highlight that the ecological importance of VOCs in ecosystems surpasses conventional perceptions.

VOCs enter the soil primarily via diffusion from aboveground emissions or direct release from roots, thereby influencing the composition and functional potential of soil microbial communities [17, 18]. Under biotic (e.g. pathogen attack) or abiotic (e.g. drought, salinity) stress, plants emit VOCs that modulate soil microbial community structure and function, thereby enhancing plant resilience to adverse conditions [19–21]. For instance, in potato–onion intercropping systems, specific VOCs such as dipropyl disulfide enrich beneficial taxa (e.g. *Bacillus* spp.) and inhibit the growth of pathogenic microorganisms (e.g. *Proteobacteria*) [18]. Such microbiome-mediated effects can enhance soil suppressiveness and improve plant stress tolerance. However, most existing work has focused on compositional outcomes, leaving open the question of how VOCs mechanistically shape microbial ecological strategies.

A major conceptual gap lies in understanding whether plant VOCs function primarily as informational signals, selective stressors, or both, and how these roles depend on environmental context. Ecological theory predicts that chemical gradients can act as filters, favoring organisms with particular life-history strategies, whereas excluding others [22, 23]. In microbial communities, such filtering is often expressed along trade-offs among competitive ability, stress tolerance, and rapid exploitation of transient resources, as formalized in the competitor–stress tolerator–ruderal (CSR) framework. Microbial responses to chemical cues do not occur in isolation but are embedded within resource landscapes that constrain physiological options. Among essential nutrients, iron occupies a central position in microbial ecology due to the low availability in soils [24–26]. Plants actively influence iron availability through root exudation and chelation, and microbes respond via siderophore production [27, 28]. Recent work suggests that volatile compounds can indirectly modify iron availability or perception [29]. This hints that nutrient limitation may gate microbial responses to plant volatiles [30, 31]. However, whether and how iron availability integrates with VOC signaling to shape microbial ecological strategies remains poorly understood.

Previously, β-caryophyllene has been identified as a key VOC that is widely present in the rhizosphere secretions of many plants, where it actively modulates the soil microbiome [32, 33]. Nevertheless, the mechanisms by which it precisely modulates soil microbial communities and drives their adaptive evolution remains poorly explored, especially regarding ecological-scale dynamics and genetic-level alterations. Evidences from early studies suggested VOCs may drive the evolution of soil microbial communities through two primary pathways: first, by altering the soil chemical environment (e.g. pH and redox state) to indirectly influence microbial activity [17, 34, 35]; second, by acting directly as signaling molecules to regulate microbial gene expression and metabolic pathways [36, 37]. For instance, in response to exposure of VOCs emitted by resistant Lima bean, the expression of defense-related genes *PR1*, *PR2*, and *PR4* was induced in the susceptible varieties [38]. Yet, these mechanistic studies still have significant limitations that hinder a holistic understanding of the functional roles of VOCs in soil ecosystems. To be exact, the molecular mechanisms underlying VOC–microbe interactions remain poorly characterized, particularly in terms of how specific VOCs selectively modulate microbial taxa and their functional attributes.

**Figure 1:**
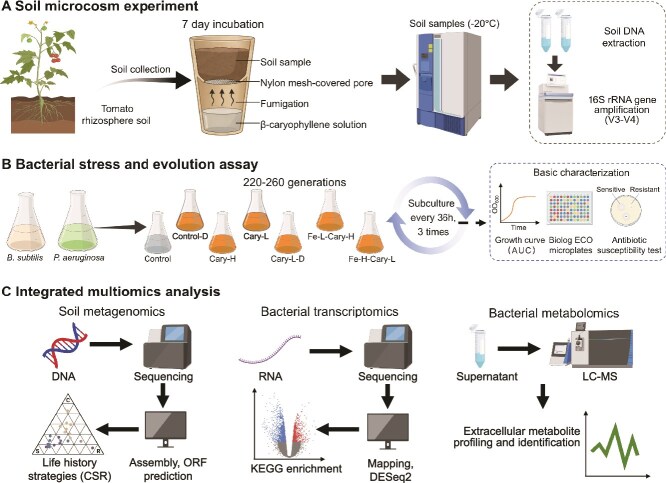
Experimental design for investigating β-caryophyllene-mediated bacterial adaptation. (A) 16S rRNA and metagenomic sequencing of soil microcosms fumigated with β-caryophyllene to assess its impact on microbial ecology. (B) Laboratory culture experiments of key strains under β-caryophyllene stimulation, with multi-omics analysis to uncover the mechanisms of adaptive evolution.

Here, we investigate how plant volatile gradients shape microbial community assembly by acting as ecological filters that select for distinct life-history strategies. Using tomato rhizosphere soil as a model system and β-caryophyllene as a representative plant volatile, we test the hypothesis that VOC concentration determines whether β-caryophyllene functions as a signal or a selective stressor, thereby reprogramming microbial competitive and ruderal strategies. We further propose that these responses may be influenced by iron availability, which could serve as an environmental factor associated with volatile perception and microbial physiological trade-offs (Fig. 1). To test these ideas, we combine soil microcosm experiments with strain-level physiological assays, integrated transcriptomic and metabolomic analyses of representative taxa. By explicitly linking chemical gradients, resource limitation, and life-history trade-offs, this study provides a mechanistic framework for understanding how plant-derived volatiles and nutrient landscapes jointly structure soil microbiomes.

## Materials and methods

### Soil microcosm experiment

Soil samples were collected from the tomato rhizosphere at the long-term tomato-cucumber rotation experimental platform in Wuqing District, Tianjin, China (117° 2′ 46″ E, 39° 32′ 5″ N) (Table S2). Microcosms were established using bamboo fiber paper cups (77 mm × 93 mm × 52 mm) as experimental containers. A 15 mm × 15 mm opening was made at the bottom of each upper cup and sealed with a nylon mesh to prevent soil loss and allow vapor-phase diffusion of β-caryophyllene into the upper soil compartment. Transparent plastic film was used to cover the joints of the apparatus to maintain a sealed fumigation environment. Two concentrations of β-caryophyllene were applied as previously described, where these concentrations significantly altered the composition and abundance of VOCs in tomato leaves: a high-concentration treatment (Cary-H), in which 40 μL of β-caryophyllene solution was added to the lower container, and a low-concentration treatment (Cary-L), with 20 μL added [39]. Although these concentrations exceed typical tissue levels under natural conditions, they were necessary to ensure reproducible biological responses in our liquid culture system due to the limited aqueous partitioning of this hydrophobic volatile. Soil samples were collected after 7 days of fumigation, immediately frozen at −20°C, and stored until further analysis.

### Bacterial growth and carbon utilization assay

The *Bacillus subtilis* (*B. subtilis*) and *Pseudomonas aeruginosa* (*P. aeruginosa*) were used in this study. These strains were selected primarily as genetically well-characterized model organisms to facilitate future mechanistic studies of β-caryophyllene–bacterial interactions. The 16S rRNA gene sequence analysis confirmed that *B. subtilis* and *P. aeruginosa* both share high sequence similarity (~96%) with the *Bacillus* and pseudomonad amplicon sequence variants (ASVs) that responded to β-caryophyllene in our mesocosm experiment, indicating close phylogenetic relatedness to the environmentally responsive bacteria in this study. The *B. subtilis* strain (CGMCC No. 33173) was originally isolated from the rhizosphere soil of tomato plants by our research group and deposited in the China General Microbiological Culture Collection Center (CGMCC) as a patented strain. The *P. aeruginosa* (CMCC (B) 10104) was obtained from the National Center for Medical Culture Collections in China (CMCC, https://www.cmccb.org.cn/htmls/index.html) as a standard reference strain. Both bacteria were cultured in Luria–Bertani (LB) medium at 37°C. The medium contained the following components (L^−1^): 10 g of tryptone, 5 g of yeast extract, 10 g of sodium chloride (NaCl), and pH 7.0. The solid LB medium also contained 20 g of agar powder. All media were sterilized at 121°C for 30 min before using. The growth curves of the strains were determined by OD600 [40–42]. Bacteria cultured under stress conditions (i.e. β-caryophyllene exposure and iron availability) were inoculated into LB liquid medium at a volume of 200 μL, and then incubated in the dark at 180 rpm and 30°C for 48 h. At 0, 2, 4, 6, 8, 12, 16, 24, 36, and 48 h, the OD600 of the samples were measured by BIO-RAD iMark™ Microplate Reader to characterize the bacterial growth.

The area under the curve to activate (AUC) of the growth curve was calculated using the trapezoidal rule with the formula [41]: 


$$ \textrm{AUC}\ = \sum_{\mathit{\mathsf{i}}=\mathsf{1}}^{\mathit{\mathsf{n}}-\mathsf{1}}\frac{\left({\mathit{\mathsf{OD}}}_{\mathit{\mathsf{i}}}+{\mathit{\mathsf{OD}}}_{\mathit{\mathsf{i}}+\mathsf{1}}\right)}{\mathsf{2}}\times \left({\mathit{\mathsf{t}}}_{\mathit{\mathsf{i}}+\mathsf{1}}-{\mathit{\mathsf{t}}}_{\mathit{\mathsf{i}}}\right) $$

Here, *OD_i_* and *OD*_*i* + 1_ are the optical density values at 600 nm measured at consecutive time points *t_i_* and *t*_*i* + 1_, respectively; *t_i_* and *t*_*i* + 1_ denote successive measurement times (in hours); *n* is the total number of time points included in the growth curve. The summation accounts for the cumulative growth over the whole experimental duration. Bacterial carbon utilization capabilities were determined using the Biolog EcoPlate assay [43]. A 10-fold dilution of the bacterial suspension was prepared using sterile water to ensure the initial concentration of the bacterial suspension was OD600 = 0.1. Biolog EcoPlate were preheated to 25°C prior to inoculation. Each well was inoculated with 150 μL of the diluted suspension using a pipette. The plates were incubated at 37°C in a constant-temperature incubator inside sealed bags to prevent desiccation. The absorbance at 590 nm and 750 nm was measured every 24 h over a period of 0 to 168 h.

### Experimental design of β-caryophyllene and iron stress on bacteria

We performed stress experiments to test the effects of β-caryophyllene and iron on bacterial resistance. The *B. subtilis* and *P. aeruginosa* strains were inoculated into 50 ml of LB medium, respectively, and darkly incubated in an orbital shaker at 180 rpm and 30°C for 24 h to revive the strains from frozen stocks. Subsequently, two concentrations of β-caryophyllene (2.26 mg/ml and 0.56 mg/ml, hereafter high and low) and FeCl_3_ (18 nM and 0.45 nM, hereafter high and low), as well as their interaction, were used to co-culture with the two bacteria. In total, there were seven treatments: only high (Cary-H) or low (Cary-L) concentration of β-caryophyllene, low concentrations of both β-caryophyllene and FeCl_3_ (Fe-L-Cary-L), low-concentration β-caryophyllene with high-concentration FeCl_3_ (Fe-H-Cary-L), low-concentration β-caryophyllene with 2,2′-Dipyridyl (Cary-L-D), Control with 2,2′-Dipyridyl (Control-D), and Control, only bacteria with no solution. Each treatment with three replicates.

In each treatment, 200 μL of the activated bacteria were added, and the samples were co-cultured under the above conditions. Then subculture every 36 h, and each subculture was supplemented with the same amount of β-caryophyllene and FeCl_3_ solution as the initial culture. A third of the bacterial culture was transferred into a new LB medium containing 50 ml of sterilized liquid for cultivation, completing a total of three subcultures. Bacterial cultures were continuously exposed to either β-caryophyllene or Fe^3+^ for 144 h prior to RNA extraction. Each subculture retained 2 ml samples in sterilized centrifuge tubes and stored at −80°C for subsequent Transcriptomic analysis.

### Antibacterial susceptibility test

To assess the effects of β-caryophyllene and FeCl_3_ stress on bacterial antibiotic resistance, we conducted the antibacterial resistance test. Two common antibiotics were used for the bacteria after the third subculture (approximately 220–260 generations) after co-cultivation with β-caryophyllene. Antibiotics were purchased from Company Liofilchem S.r.l., and included Norfloxacin (NOR), Tetracycline (TET). Two antibiotic susceptibility papers were placed on LB medium inoculated with the third-generation strains and cultured at 37°C for 24 h. Subsequently, we measured the diameter around each antibiotic paper to determine the sensitivity of *B. subtilis* and *P. aeruginosa* to antibiotics under different treatments [44, 45].

### Bacterial community composition analysis

The total genomic DNA of each sample was extracted from 0.5 g homogenized soil using a MagBeads FastDNA Kit for Soil (MP Biomedicals, CA, USA) following the manufacturer’s instructions. The V3-V4 region of the bacterial 16S rRNA was amplified using the primer pairs 338F (5′-ACTCCTACGGGAGGCAGCAG-3′) and 806R (5′-GGACTACNNGGGTATCTAAT-3′) [46]. The purified PCR products were cleaned up using BECKMAN AMPure XP beads, and sequencing libraries were prepared using the TruSeq Nano DNA LT Kit (Illumina). The bioinformatics analysis of the raw sequences was carried out using the DADA2 package via the dadasnake pipeline to infer ASVs. Briefly, the process involved primer trimming with cutadapt, quality filtering, denoising, read merging, and chimera removal. The taxonomic assignment of bacterial ASVs was performed using the SILVA database (v138).

### Metagenomic analysis

Genomic libraries were constructed from 1 ng DNA using the NEBNext Ultra DNA Library Prep Kit (NEB, USA), following the manufacturer’s protocol, and sequenced on a NovaSeq 6000 System (Illumina, USA) with a paired-end strategy. Raw reads were subjected to quality control with Readfq (V8) to discard low-quality sequences (≥10% N bases or >50% of bases with Q ≤ 10). Subsequent denovo assembly was performed using SOAPdenovo (v2.04). Unmapped paired-end reads were merged and reassembled to improve continuity. Contigs longer than 500 bp were retained for open reading frame (ORF) prediction, and a nonredundant gene catalog was constructed from ORFs exceeding 100 nt in length. Taxonomic assignment of predicted genes was conducted with DIAMOND (v0.9.9) against the NCBI Non-Redundant Protein Database (version 2018.01). Antibiotic resistance genes (ARGs) were identified by alignment to the Comprehensive Antibiotic Resistance Database (CARD) using an e-value cutoff of 1 × 10^−30^. Gene abundance was normalized and reported in transcripts per million (TPM) to enable cross-sample comparability of expression levels.

### Microbial life-history strategy analyses

To elucidate the multivariate axes underpinning bacterial life-history strategies, we applied Multivariate Co-inertia Analysis (MCOA) to integrate functional profiles from eggNOG, Kyoto Encyclopedia of Genes and Genomes (KEGG), and CAZy databases within the CSR framework [47]. We evaluated the associations among ARGs, eggNOG, and CAZy functional genes, and evolutionary traits. Specifically, the competitive (C) strategy was represented by the abundance of dominant ARGs, the stress-tolerator (S) strategy by glycoside hydrolase (GH), auxiliary activities (AA) gene expression, and the ruderal (R) strategy by nitrogen-related motility gene expression [48].

### Transcriptomic analysis

All treatments (i.e. Control, Cary-H, Cary-L, Fe-L-Cary-L, Fe-H-Cary-L, Control-D, and Cary-L-D) were centrifuged at 10 000 rpm for 2 min to collect cells. Total RNA was isolated using the Trizol Reagent (Invitrogen Life Technologies). Quality and integrity were determined using a NanoDrop spectrophotometer (Thermo Scientific) and a Bioanalyzer 2100 System (Agilent). The sequencing library was then sequenced on NovaSeq 6000 System (Illumina) by Shanghai Personal Biotechnology Cp. Ltd. Quality metrics for the raw FASTQ data were calculated, and the raw data were then filtered using fastp (v0.22.0). Reads with an average quality score lower than Q20 were removed to obtain clean data, which were used for all subsequent analyses.

The filtered reads were aligned to the reference genome using Bowtie2 (version 2.5.1). HTSeq (version 0.9.1) was employed to count the gene read count values, which were considered as the initial gene expression levels. To enable comparison of gene expression levels across different genes and samples, the Fragments Per Kilobase of exon per Million fragments mapped (FPKM) method was used for normalization. Differentially expressed mRNAs were identified using DESeq (version 1.38.3), transcripts with |log₂FoldChange| >1 and a *P*-value <.05 were regarded as differentially expressed. TopGO was used for Gene Ontology (GO) enrichment analysis of the differentially expressed genes (DEGs). The *P*-values were calculated using the hypergeometric distribution method, and GO terms with significant enrichment (*P*-value <.05) were identified to infer the main biological functions of the DEGs. The ClusterProfiler software (version 4.6.0) was utilized for KEGG pathway enrichment analysis of the DEGs. Pathways with significant enrichment (*P*-value <.05) were highlighted. DEGs were functionally annotated by aligning predicted protein sequences against the CARD using DIAMOND blastx, and functional annotations were assigned based on the best-matching hits.

### Quantification of siderophore production

Siderophore production was quantified using the Chrome Azurol S (CAS) assay. Briefly, 121 g of CAS liquid medium base was dissolved in 900 ml deionized water and sterilized at 115°C for 20 min. After cooling to 50–60°C, 100 ml sterile 10× buffer solution preheated to 60°C was added aseptically. Bacterial strains were inoculated into the medium and incubated at 28°C with shaking at 180 rpm for 2–3 d. Following incubation, cultures were centrifuged at 10 000 rpm for 10 min, and the supernatants were collected. Equal volumes of culture supernatant and 2 × CAS detection solution (prepared by diluting 10× CAS reagent with sterile water at a 1:4 ratio) were mixed and incubated in the dark for 30 min. Absorbance was measured at 630 nm (*A_s_*). Uninoculated medium mixed with CAS detection solution was used as the reference control (*A_r_*). Siderophore production was calculated as siderophore units (SU) according to the following equation:


$$ \mathrm{SU}\left(\%\right)=\frac{A_r-{A}_s}{A_r}\times 100\% $$

where *A_r_* is the absorbance of the reference control and *A_s_* is the absorbance of the sample.

### Metabolomic analysis

The cultured bacterial solution was centrifuged at 10 000 rpm for 4 min, with 2 ml of the supernatant being taken as an experimental sample for measuring extracellular metabolites. Thaw the experimental sample at 4°C, vortex the sample for 1 min after thawing, and mix evenly. Accurately transfer an appropriate amount of sample into a 2 ml centrifuge tube, concentrate and dry it. Add 500 μL methanol solution into the sample tube dried and vortex for 1 min. Centrifuge for 10 min at 12 000 rpm and 4°C, take all the supernatant, transfer it to a new 2 ml centrifuge tube, concentrate and dry it. The sample tube was added with 150 μL of a 2-amino-3-(2- chlorophenyl) propionic acid solution (4 ppm) prepared in 80% methanol water to redissolve the sample. The supernatant was then filtered through a 0.22 μm filter membrane and transferred into the detection bottle for liquid chromatograph mass spectrometer (LC–MS) detection [49].

### Statistical analysis

The Bray–Curtis dissimilarity matrix was calculated and the β-diversity of the community was analyzed by principal component analysis (PCA). Permutational multivariate analyses of variance (PERMANOVA) were used to examine the difference in community structure. Alpha diversity was calculated based on the relative abundance of bacterial genera using the “vegan” package. One-way ANOVA was used to assess the significant differences among different treatments followed by Tukey’s HSD *post hoc* tests. DEGs were identified using the Personalbio GeneSCloud platform (https://www.genescloud.cn/home) with significance defined as |log₂(fold change)| > 1 and adjusted *P*-value <.05 (Benjamini–Hochberg correction). Volcano plots of DEGs were generated with “ggplot2” package (version 4.3.3). Spearman correlation coefficients were analyzed using “linkET,” “ggplot2,” “dplyr,” and “cols4all” packages. Redundancy analysis (RDA) was performed using Canoco 5 to explore multivariate relationships between DEGs and phenotypic variables. The stacked bar charts created using Origin (2021) visually presented the composition of soil microbial communities and the distribution of ARGs in *B. subtilis* and *P. aeruginosa* under different treatment conditions; line charts were created to show the bacterial growth dynamics and changes in carbon utilization ability at different time points.

## Results

### β-Caryophyllene alters soil bacterial community structure and life-history strategies

**Figure 2:**
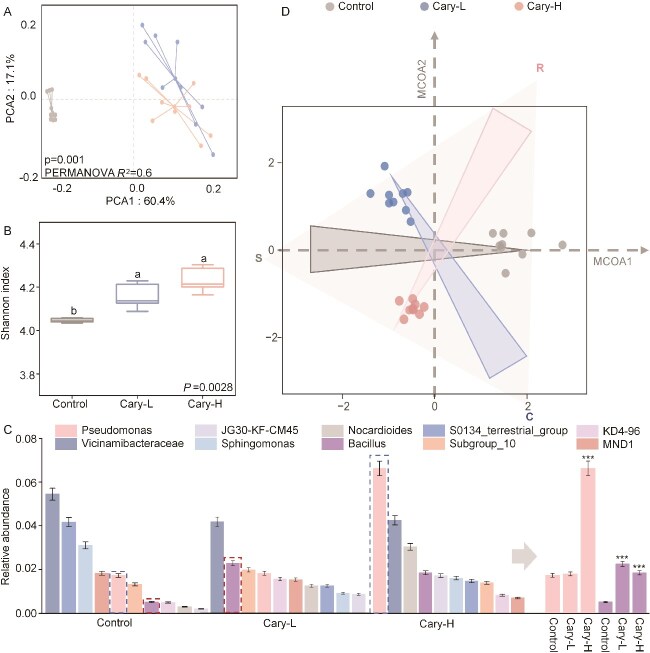
Changes in the structure and evolutionary strategies of soil bacterial community under β-caryophyllene stress. (A) PCA of bacterial communities based on different concentration of β-caryophyllene stress. (B) Comparison of bacterial Shannon index under different concentrations of β-caryophyllene stress. (C) Changes in the relative abundance of 10 dominant bacterial genera after β-caryophyllene stress. (D) The life-history strategies (C-S-R model) for the evolution of soil bacteria in different treatments. The C, S, and R mean competitor, stress tolerant, and ruderals, respectively. Control: soil without β-caryophyllene fumigation. Cary-H: soil with high concentration of β-caryophyllene fumigation. Cary-L: soil with low concentration of β-caryophyllene fumigation. The lowercase letters represent differences among treatments (*P* < .05). ^***^  *P* < .001.

To determine how plant VOCs influence microbial community assembly, we exposed tomato rhizosphere soil microcosms to low and high concentrations of β-caryophyllene. We found that exposure to β-caryophyllene significantly restructured soil bacterial communities (Fig. 2). PCA of 16S rRNA profiles revealed a clear separation among control, low-concentration (Cary-L), and high-concentration (Cary-H) treatments, with the first two axes explaining 77.5% of total community variation (PERMANOVA: *P* < .05; Fig. 2A). The Cary-L treatment produced the greatest compositional divergence, indicating that β-caryophyllene exerts strong ecological effects even at sub-stress concentrations.

Consistent with these compositional shifts, β-caryophyllene increased bacterial alpha diversity at both concentrations relative to the control (Fig. 2B). This indicates that volatile exposure altered competitive hierarchies rather than uniformly suppressing microbial growth. Community composition analysis revealed pronounced, concentration-dependent shifts in dominant taxa (Fig. 2C). Cary-L selectively enriched 3.2-flod *Bacillus* spp., whereas Cary-H favored *Pseudomonas* spp., whose relative abundance increased approximately 2.8-fold. Gram-positive *Bacillus* and gram-negative *Pseudomonas* represent phylogenetically and functionally distinct bacterial lineages emerged as key responders to β-caryophyllene exposure. These different bacterial taxa suggest that β-caryophyllene selects among contrasting ecological strategies rather than individual species perse.

Mapping these community shifts onto a life-history strategy framework revealed systematic changes in ecological strategies (Fig. 2D). Control communities were dominated by stress-tolerant (S-strategy) taxa, reflecting adaptation to the baseline soil environment. In contrast, Cary-L communities shifted toward a competitive (C-strategy) profile, consistent with the enrichment of *Bacillus*, a genus associated with antagonism and resource monopolization. High β-caryophyllene concentrations instead promoted ruderal (R-strategy) communities dominated by fast-growing opportunists such as *Pseudomonas* (Fig. S1). Together, these results show that β-caryophyllene acts as a concentration-dependent ecological filter that reorganizes microbial communities along defined life-history axes.

### Contrasting physiological strategies underlie competitive and ruderal responses to β-caryophyllene

**Figure 3:**
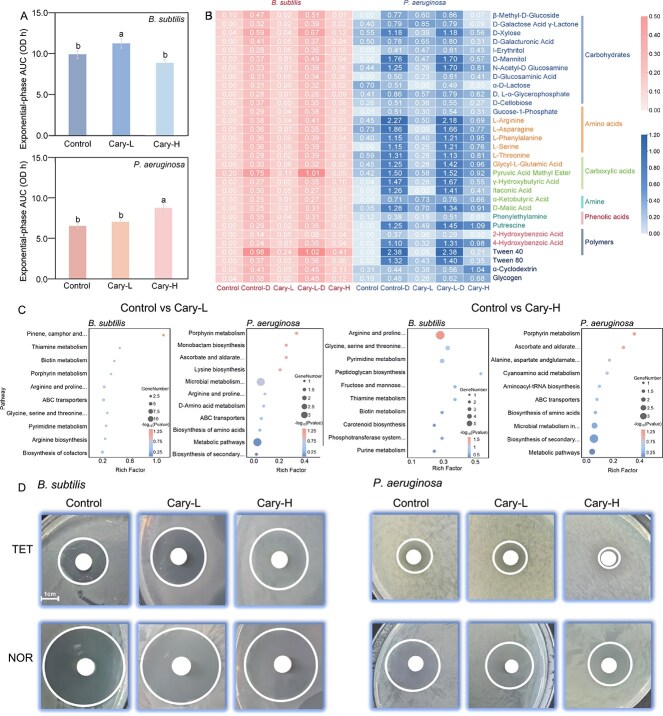
The phenotypic responses of *B. subtilis* and *P. aeruginosa* to different concentrations of β-caryophyllene stimulation. (A) AUC of the growth curves of *B. subtilis* and *P. aeruginosa* with β-caryophyllene co-culture. (B) The utilization spectrum of 31 common rhizosphere carbon sources by *B. subtilis* and *P. aeruginosa* under β-caryophyllene stimulation, with absorbance per well indicating utilization intensity. (C) KEGG enrichment pathways of differential metabolites in *B. subtilis* and *P. aeruginosa* under β-caryophyllene co-culture. (D) Changes in the inhibitory zone size of tetracycline and norfloxacin for *B. subtilis* and *P. aeruginosa* after co-culture with β-caryophyllene. The lowercase letters represent differences among treatments (*P* < .05).

To uncover the mechanistic basis of these community-level shifts, we selected *B. subtilis* as representative competitive strategists and *P. aeruginosa* as representative ruderal strategists. These strains share high sequence similarity with β-caryophyllene-responsive ASVs identified in soil communities (Table S1). Exposure to β-caryophyllene significantly altered growth dynamics in both species (Fig. 3A; Fig. S2). In *B. subtilis*, low β-caryophyllene concentrations increased the maximum specific growth rate and reduced lag time (*P* < .05), consistent with enhanced competitive performance under moderate environmental modification. In *P. aeruginosa*, both low and high concentrations markedly shortened the lag phase (by ~25%) and stimulated exponential growth, reflecting a ruderal strategy favoring rapid population expansion.

Phenotypic microarray analysis of 31 rhizosphere-relevant carbon sources further revealed that β-caryophyllene reshaped metabolic niche breadth in a taxon-specific manner (Fig. 3B). *B. subtilis* preferentially expanded its utilization of amino acids and polymeric substrates, reflecting a competitive strategy focused on efficient resource acquisition and sustained growth. Conversely, *P. aeruginosa* displayed broader enhancement of amine and amino acid utilization, consistent with metabolic flexibility and opportunistic resource use.

Metabolomic profiling corroborated these physiological patterns. In *B. subtilis*, β-caryophyllene exposure upregulated arginine and proline metabolism, with accumulation of intermediates such as N-acetylglutamate semialdehyde and guanidinoacetate (Fig. 3C). In *P. aeruginosa*, enhanced alanine, aspartate, and glutamate metabolism, alongside increased L-asparagine biosynthesis, dominated the metabolic response. These distinct metabolic trajectories indicate that β-caryophyllene does not elicit a uniform stress response but instead amplifies preexisting strategy-specific physiological programs.

These physiological shifts were accompanied by changes in antibiotic susceptibility (Fig. 3D). In *B. subtilis*, β-caryophyllene increased sensitivity to tetracycline but reduced sensitivity to norfloxacin, whereas *P. aeruginosa* exhibited decreased susceptibility to both antibiotics. These contrasting responses align with their inferred life-history strategies, indicating that VOC-driven ecological filtering is executed through coordinated trade-offs among growth, metabolism, and defense.

### β-Caryophyllene reprograms transcriptional networks underlying metabolic and defensive trade-offs

**Figure 4:**
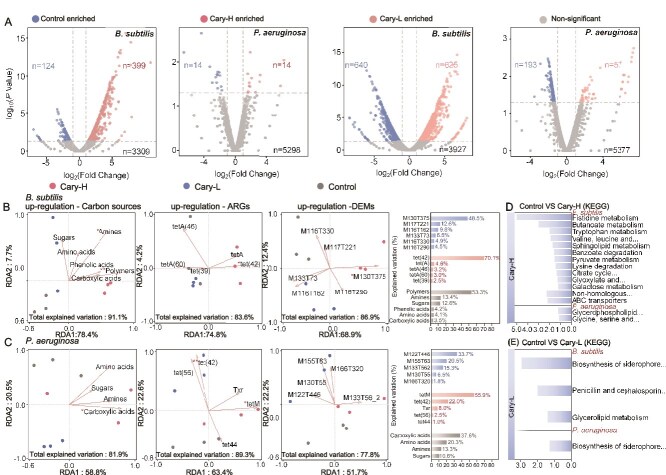
Linkage between DEGs and the phenotypic changes in *B. subtilis* and *P. aeruginosa*. (A) The volcano plots of DEGs in *B. subtilis* or *P. aeruginosa* for the comparisons of Control vs. Cary-H and Control vs. Cary-L. (B) RDA between carbon utilization types, ARGs, differential metabolites, and DEGs in *B. subtilis*. (C) RDA between carbon utilization types, ARGs, differential metabolites, and DEGs in *P. aeruginosa*. (D) The KEGG enrichment pathways of DEGs in *B. subtilis* and *P. aeruginosa* under high-concentration β-caryophyllene stimulation (Cary-H). (E) The KEGG enrichment pathways of DEGs in *B. subtilis* and *P. aeruginosa* under low-concentration β-caryophyllene stimulation (Cary-L).

To link physiological responses to molecular mechanisms, we profiled transcriptomic responses of *B. subtilis* and *P. aeruginosa* under low and high β-caryophyllene exposure. Both species exhibited concentration-dependent transcriptional responses, but the scale and structure of these responses differed markedly. In *B. subtilis*, Cary-L induced extensive transcriptional remodeling, with 625 upregulated and 640 downregulated genes relative to the control, exceeding the response observed under Cary-H (Fig. 4A). This suggests that low-dose β-caryophyllene acts primarily as a regulatory signal rather than a stressor. In contrast, *P. aeruginosa* exhibited a pronounced response under Cary-L but minimal differential expression under Cary-H, consistent with a shift from active adaptation to passive tolerance at high concentrations.

RDA revealed strong coupling between transcriptional changes and phenotypic outcomes (Fig. 4B and C). In *B. subtilis*, DEGs explained over 90% of the variance in carbon utilization patterns and over 80% of the variance in ARG expression and metabolite profiles. Polymeric carbon utilization and the metabolite N-acetyl-L-glutamate 5-semialdehyde emerged as dominant drivers. In *P. aeruginosa*, DEGs similarly accounted for most of the observed differences in carbon use (>80%), resistance traits (~90%), and metabolic outputs (>90%) with carboxylic acid metabolism and *tetM* genes as major contributors.

KEGG pathway enrichment revealed concentration-dependent transcriptional programs (Fig. 4D and E). In *B. subtilis*, Cary-L preferentially enriched pathways related to secondary metabolism, membrane biosynthesis, and iron acquisition, whereas Cary-H emphasized amino acid metabolism and DNA repair (Fig. 4D). In *P. aeruginosa*, Cary-L strongly upregulated siderophore biosynthesis, whereas Cary-H showed limited pathway enrichment (Fig. 4E). These patterns indicate that low-dose β-caryophyllene elicits active physiological reprogramming, whereas high-dose exposure constrains regulatory flexibility, particularly in ruderal strategists.

Based on this, we further quantified siderophore production in the two bacterial strains (Fig. S3). The results showed that, compared with the Control treatment, Cary-L significantly increased siderophore production, which was consistent with the transcriptomic results showing upregulation of genes involved in siderophore biosynthesis. Furthermore, under iron-limited conditions (Control-D and Cary-L-D), all strains exhibited higher levels of siderophore secretion, indicating that external iron deprivation further activated bacterial iron acquisition responses. Bacteria exposed to β-caryophyllene treatment displayed the highest siderophore production under iron-limited conditions (~65%), which was significantly higher than that of the other treatments. These results suggest that β-caryophyllene treatment induced a high iron-demand state in the bacteria.

### Iron availability gates microbial responses to low-dose β-caryophyllene

**Figure 5:**
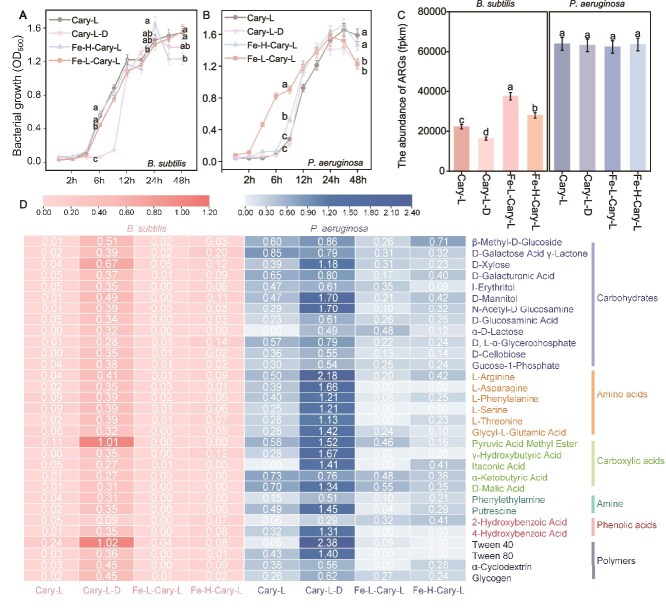
Bacterial physiological phenotype after co-culture with Fe^3+^ and β-caryophyllene. (A) The growth curve of *B. subtilis* after cultivation with β-caryophyllene and Fe^3+^. (B) The growth curve of *P. aeruginosa* after cultivation with β-caryophyllene and Fe^3+^. (C) Expression of ARGs in *B. subtilis* or *P. aeruginosa* after cultivation with β-caryophyllene and Fe^3+^. (D) The utilization spectrum of 31 common rhizosphere carbon sources by *B. subtilis* and *P. aeruginosa* under β-caryophyllene and Fe^3+^ stimulation, with absorbance per well indicating utilization intensity. The lowercase letters represent differences among treatments (*P* < .05).

Across both taxa, low-dose β-caryophyllene consistently induced siderophore biosynthesis pathways, suggesting that iron availability acts as a key environmental integrator of volatile responses. To test this, we manipulated iron availability under Cary-L conditions and examined resulting physiological and functional shifts. Under iron-limited conditions (Cary-L-D), the lag phase of both two bacterial strains was significantly prolonged (by 25% ~ 50%). Iron (Fe^3+^) supplementation under Cary-L conditions markedly altered growth dynamics in both species (Fig. 5A and B). In *B. subtilis*, iron addition produced a diauxic growth pattern, indicating sequential resource utilization. In *P. aeruginosa*, Fe^3+^ significantly shortened lag phase and increased exponential growth rate in a dose-dependent manner (*P* < .05), reinforcing its ruderal strategy.

Iron availability also reshaped defensive investment. In *B. subtilis*, iron supplementation restored and significantly increased ARG expression relative to Cary-L alone (Fig. 5C), demonstrating a reversible trade-off between iron acquisition and antibiotic resistance. In *P. aeruginosa*, ARG expression remained largely unchanged, suggesting consistent with its intrinsic tolerance strategy and reduced reliance on iron-mediated regulation.

Carbon utilization patterns further revealed strategy-specific integration of volatile and resource cues (Fig. 5D). In *B. subtilis*, iron supplementation shifted metabolism toward simple sugars at the expense of amino acids and polymers, whereas *P. aeruginosa* reduced utilization of nitrogen-rich substrates. Under iron-limited conditions, both two bacterial strains exhibited enhanced global carbon source utilization, suggesting that they increase substrate utilization breadth and metabolic flexibility to improve adaptation to resource-limited environments. Together, these results demonstrate that iron availability functions as a decisive environmental switch that gates microbial responses to β-caryophyllene. By modulating growth, metabolism, and defense, iron determines whether β-caryophyllene acts as a signaling cue that primes competitive strategies or as a selective pressure favoring ruderal traits.

## Discussion

**Figure 6:**
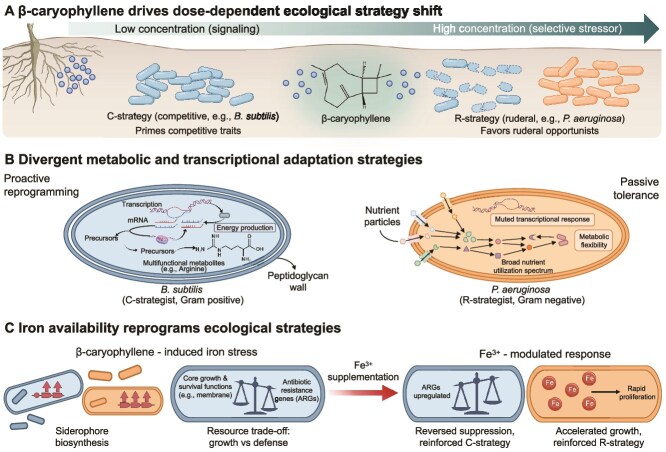
Multi-perspective mechanism diagram of soil microbial responses to β-caryophyllene.

Plant VOCs are increasingly recognized as powerful, yet underappreciated, drivers of belowground ecology [31, 50]. By integrating community-scale experiments with strain-level physiology and multi-omics analyses, this study reveals how a single, ubiquitous plant sesquiterpene, β-caryophyllene, can steer microbial community assembly and adaptive trajectories through a hierarchical and context-dependent mechanism (Fig. 6). Our findings establish three general principles: (i) VOCs impose concentration-dependent ecological filtering, (ii) microbial responses are executed through life-history strategy specific metabolic and transcriptional programs, and (iii) nutrient availability, particularly iron, acts as a central environmental integrator that gates volatile-mediated outcomes.

β-Caryophyllene functions as a bifunctional cue, where concentration dictates its ecological role. At low concentrations, it acts as a signaling molecule that primes competitive (C-strategy) traits, whereas at high concentrations, it transitions into a selective stressor that favors ruderal (R-strategy) opportunists, thereby driving concentration-dependent ecological filtering. Specifically, our findings demonstrate that β-caryophyllene restructures community hierarchies by modulating keystone genera, thereby altering the balance of life-history strategies. Under high-concentration stress, the microbial community shifted towards an R strategy, focusing on rapid reproduction to occupy niches left by extinct stress-sensitive taxa. This shift was associated with the increased Shannon index under high-concentration β-caryophyllene treatment, which was primarily attributed to the expansion of opportunistic taxa as a result of the reduction in sensitive species. Plant-derived β-caryophyllene can drive directional community assembly by dose-dependently selecting for taxa with pre-adapted ecological strategies. The divergent responses of *Pseudomonas* (gram-negative) and *Bacillus* (gram-positive) further illustrate how phylogenetic traits, particularly cell envelope architecture, determine baseline tolerance and shape adaptive trajectories along the β-caryophyllene concentration gradient.

This ecological shift is underpinned by extensive and divergent metabolic reprogramming. The induced transcriptional changes dominantly explain phenotypic variance (e.g. 90% of carbon utilization), steering *B. subtilis* toward polymer/amino acid metabolism and *P. aeruginosa* toward carboxylic acid metabolism, directly linking gene expression to life-history strategy execution. *B. subtilis* exhibited a competitive strategy, channeling resources into the biosynthesis of multifunctional metabolites such as arginine—a dual carbon and nitrogen source that supports protein synthesis and energy homeostasis under stress [51, 52]. This metabolic specialization reflects an investment in efficiency and resilience, typical of competitors in stable or moderately perturbed environments. In contrast, *P. aeruginosa* adopted a ruderal strategy, characterized by broad catabolic versatility rather than reliance on any single substrate. Its capacity to rapidly utilize diverse carbon and nitrogen sources—including L-asparagine and glycyl-L-glutamic acid—enables opportunistic exploitation of transient nutrient generated by volatile-induced community turnover. Thus, the metabolic-level specialization provides a mechanistic basis for microbial adaptation to β-caryophyllene stimulation.

Transcriptomic analyses indicate that β-caryophyllene induces dose-dependent and taxon-specific regulatory responses, consistent with its dual function as a signal at low concentrations and a stressor at high concentrations. In *B. subtilis*, low-dose exposure (Cary-L) triggered extensive transcriptional reprogramming, exceeding that observed under high-dose treatment, reflecting a proactive reallocation of resources toward essential biosynthetic and maintenance processes, whereas repressing non-essential functions [53]. In contrast, *P. aeruginosa* displayed a distinct concentration-dependent strategy: Cary-L elicited an active adaptive response consistent with environmental sensing, whereas Cary-H resulted in minimal transcriptional changes relative to the control. This muted response likely reflects a shift from active adaptation at low doses to a passive tolerance state under acute stress, characterized by global suppression of gene expression.

The changes in antibiotic susceptibility further indicate that β-caryophyllene can reshape microbial physiological states and environmental adaptation strategies. This response may be driven by its multi-level physiological regulation. It may alter cell membrane permeability, thereby affecting antibiotic influx, and regulate the expression of efflux pump systems, thus modifying active antibiotic efflux. In addition, β-caryophyllene may influence iron availability or iron acquisition-related regulatory networks, indirectly reshaping cellular energy metabolism and redox balance, which in turn modulates antibiotic-related metabolic processes. Differences among bacterial species in membrane structure, efflux capacity, and metabolic strategies may collectively drive their divergent responses to antibiotics, ultimately reflecting physiological trade-offs under β-caryophyllene exposure.

We identify iron competition as a central modulator of volatile-mediated interactions. Building upon the known role of root exudates in modulating iron availability [54], low-dose β-caryophyllene consistently upregulated siderophore biosynthesis in both *B. subtilis* and *P. aeruginosa*, indicating that bacterial perception of the volatile is gated by iron availability. This iron stress imposed physiological trade-offs: in *B. subtilis*, growth- and membrane-related pathways were upregulated, whereas ARGs were suppressed—a suppression that was reversed by Fe^3+^ supplementation, suggesting that iron availability influences metabolic regulation. Iron abundance also reprogrammed life-history strategies: *B. subtilis* leveraged it to reinforce long-term competitiveness (C-strategy), whereas *P. aeruginosa* favored rapid proliferation (R-strategy), sacrificing ultimate biomass. Thus, the ecological impact of β-caryophyllene is not absolute but context-dependent, with iron availability potentially influencing whether the volatile is perceived as a competitive signal or a stressor associated with ruderal traits.

In summary, we propose a general framework in which plant VOCs shape microbial community assembly and adaptation through the interaction of signal concentration and environmental context, with iron availability acting as an important mechanistic factor. By linking volatile perception to life-history strategies and resource competition, this study provides a unifying ecological and molecular basis for understanding volatile-mediated microbiome dynamics across soil ecosystems. Experiments were conducted at 37°C to ensure robust growth and metabolic activity of the model strains during the 144 h incubation. Although this temperature does not reflect natural soil conditions, it enabled controlled dissection of bacterial responses to β-caryophyllene. Caution is, therefore, warranted when extrapolating these findings to rhizosphere or soil ecosystems. Future studies in plant-associated systems will be needed to validate their ecological relevance.

## Supplementary Material

Supplementary_material_wrag181

## Data Availability

All sequence data are available in the Sequence Read Archive (SRA) under BioProject accession PRJNA1466407. All other data supporting the conclusions of this study are available within the paper.
