# Supplementary material for: β-Caryophyllene gradients act as ecological filters shaping microbial life-history strategies via iron competition
Source: ISME J. 2026 Jul 10;20(1):wrag181. doi: 10.1093/ismejo/wrag181 (PMC13477218; doi:10.1093/ismejo/wrag181)
Supplement: Supplementary file 1 [file supplementary_material_wrag181.zip › Supplementary material_wrag181.docx]

**Supplementary Information**

**β-caryophyllene gradients act as ecological filters shaping microbial life-history strategies via iron competition**

**Running title:** β-caryophyllene shapes microbiome

**Ruolan Li^a, 1^, Yuhui Li^a, 1^, Qifan Yang^a^, Yinuo Ding^a^, Gaofei Jiang^b^*, Yan Xu^a^***

a. Agro-Environmental Protection Institute, Ministry of Agriculture and Rural Affairs, Tianjin 300191, China.

b. Jiangsu Provincial Key Lab for Organic-based fertilizer development and its manipulation on soil health, Key Lab of Organic-Based Fertilizers of China, Jiangsu Collaborative Innovation Center for Solid Organic Wastes, Educational Ministry Engineering Center of Resource-saving fertilizers, Nanjing Agricultural University, Nanjing, 210095, China

^*^Corresponding author. Yan Xu, Agro-Environmental Protection Institute, Ministry of Agriculture and Rural Affairs, Tianjin 300191, China. E-mail: xuyan@caas.cn; Gaofei Jiang, Jiangsu Provincial Key Lab for Organic-based fertilizer development and its manipulation on soil health, Key Lab of Organic-Based Fertilizers of China, Jiangsu Collaborative Innovation Center for Solid Organic Wastes, Educational Ministry Engineering Center of Resource-saving fertilizers, Nanjing Agricultural University, Nanjing, 210095, China. E-mail: gjiang@njau.edu.cn.

^1^Ruolan Li and Yuhui Li contributed equally to this work.

**Fig. S1 The column showed the relative abundance of *Pseudomonas* spp. and *Bacillus* spp. in different treatments.**

**Fig. S2 The growth curve of *B. subtilis* and *P. aeruginosa* with β-caryophyllene co-culture. The lowercase letters represent differences among treatments (P < 0.05).**

**Fig. S3 Siderophore production in Bacillus subtilis, Pseudomonas aeruginosa.**

| Model strains | Species | Score | Expect | Identities | Gaps | Strand |
| --- | --- | --- | --- | --- | --- | --- |
| *Pseudomonas aeruginosa* | *Pseudomonas furukawaii* | 2607 bits (1315) | 0 | 1382/1403 (98%) | 1/1403 (0%) | Plus/Plus |
|  | *Pseudomonas cuatrocienegasensis* | 1592 bits (803) |  | 900/927 (97%) | 4/927 (0%) |  |
|  | *Pseudomonas composti* | 1631 bits (823) |  | 905/927 (97%) | 4/927 (0%) |  |
|  | *Pseudomonas xanthomarina* | 1624 bits (819) |  | 905/927 (97%) | 5/927 (0%) |  |
|  | *Pseudomonas borbori* | 2366 bits(1281) |  | 1412/1475(96%) | 10/1475(0%) |  |
|  | *Pseudomonas oleovorans* | 2498 bits (1260) |  | 1408/1456 (96%) | 1/1456 (0%) |  |
|  | *Pseudomonas stutzeri* | 2531 bits (1277) |  | 1441/1489 (96%) | 5/1489 (0%) |  |
| *Bacillus subtilis* | *Bacillus firmus* | 1703 bits (859) |  | 989/1027 (96%) | 4/1027 (0%) |  |
|  | *Bacillus aquimaris* | 1788 bits (902) |  | 1036/1076 (96%) | 4/1076 (0%) |  |
|  | *Bacillus kochii* | 1584 bits (799) |  | 914/948 (96%) | 7/948 (0%) |  |
|  | *Bacillus cohnii* | 1691 bits (853) |  | 974/1009 (96%) | 4/1009(0%) |  |

**Table S1 The sequence alignment results between the selected mode bacteria and β-caryophyllene-responsive ASVs.**

**Table S2: The physicochemical properties of the soil used in the soil microcosm experiment.**

| WSOC  (mg/g) | SOC  (g/kg) | TN  (mg/g) | NH4-N  (mg/g) | NOx-N  (mg/g) | TP  (mg/g) | AP  (mg/g) | MBC  (mg/kg) | MBN  (mg/kg) | SMC  (%) | pH | EC  (μS/cm) |
| --- | --- | --- | --- | --- | --- | --- | --- | --- | --- | --- | --- |
| 0.797 | 13.642 | 1.884 | 0.022 | 0.025 | 0.403 | 0.014 | 64.533 | 56.023 | 0.006 | 7.570 | 147.100 |
